# Supplementary material for: Green Processing, Germinating and Wet Milling Brown Rice (Oryza sativa) for Beverages: Physicochemical Effects
Source: Foods. 2020 Jul 29;9(8):1016. doi: 10.3390/foods9081016 (PMC7466225; doi:10.3390/foods9081016)
Supplement: Supplementary file 1 [file foods-09-01016-s001.pdf]

Table 1. Optimized "free-flowing" processing parameters for preparing freshly dehulled sprouted, softened, wet milled, gelatinized and enzyme treated brown rice beverages. [Previously published, (Beaulieu et al., 2020); https://onlinelibrary.wiley.com/doi/10.1002/fsn3.1534].

Processing Steps	Treatment Code	Conditions	Optimized Method
Controls	WR or BRR [†]	White rice, or freshly dehulled brown Rondo rice	Not applicable
Rinsing	Rinses [‡]	Ambient temperature, or designated per below	30 min
Soaking		Temperature	35 °C
		Temperature of added H_20	35 °C
		Time	24 hours
		Ratio (rice:water, g/g)	1:1
		Rinsed, replaced	Every 4 hours
Sprouting	GBR	Temperature	35 °C
		Rinsed	Every 4 hours
		Temperature rinse H_20	35 °C
		Time	24 hours
Softening	(none)	Temperature	70 °C max
		Temperature of added H_20	< 75 °C
		Times [§]	60 to 120 min
		Ratio (rice:water, wt/wt) [¶]	1:2
Wet Milling	PWM	Temperature	~65 °C (70 °C max)
		Temperature of added H_20	~< 75 °C
		Milling time	2 min
		Sum ratio (rice:water)	1:4
Gelatinization	Gelat	Temperature	80 °C
Enzymes & Dosage	PNZ	α-amylase	300 µL / 100 g starch
		Glucoamylase	300 µL / 100 g starch

[†] Acronyms for treatments are: WR, white rice; BRR, brown Rondo rice; GBR, germinated brown rice; PWM, post wet milling and PNZ, post saccharification enzymes. [‡] Rinses included: Water rinse (Rinse); 30 and 300 ppm peracetic acid. [§] Trial times between 60-120 min due to differences in volumes, beaker sizes, number of units run simultaneously and differences in heat energy transfer to soften kernels, as based upon a subjective softness test with a stainless steel spatula on a stainless steel table. [¶] Considered on a dry weight basis (dwb) even though kernels had absorbed water weight. Based upon original grams rice to grams water utilized.

Table 2. Informal sensory appraisal[(†)] of four anonymous commercial rice beverages (CRB).

Sensory category	Descriptor	CRB#1 BR ‡, not fortified			CRB#2 Sprouted, fortified			CRB#3 BR, fortified			CRB#4 BR, fortified		
		**Average**		**stdev**	**Average**		**stdev**	Average		stdev	Average		stdev
Aroma	Pleasant	2.25	±	0.50	1.50	±	0.58	2.50	±	0.58	2.00	±	0.63
	Nutty	1.40	±	0.89	1.80	±	0.84	1.33	±	0.52	1.20	±	0.45
	Carmel	1.33	±	0.52	1.00	±	---	1.17	±	0.41	1.00		---
Taste	Starchy	1.67	±	0.52	1.83	±	0.41	1.60	±	0.55	1.67	±	0.82
	Sweetness	2.29	±	0.76	1.33	±	0.52	2.29	±	0.76	2.57	±	0.53
	Beany	1.50	±	0.84	1.60	±	0.89	1.40	±	0.55	1.67	±	0.82
	Hay	1.00	±	0.00	1.00	±	---	1.20	±	0.45	1.17	±	0.41
	Salty	1.50	±	0.84	1.33	±	0.52	1.40	±	0.55	1.00		---
	Sour/Bitterness	1.40	±	0.55	1.33	±	0.52	1.20	±	0.45	1.50	±	0.84
	Grassy	1.00		---	1.20	±	0.45	1.20	±	0.45	1.00		---
Mouthfeel texture	Chalkiness	1.00	±	0.00	1.60	±	0.55	1.40	±	0.55	1.50	±	0.84
	Mouth coating	1.67	±	0.52	1.00	±	0.00	1.33	±	0.52	1.50	±	0.84
	Viscous	1.00	±	0.00	1.33	±	0.52	1.33	±	0.52	1.17	±	0.41
	Creamy	1.40	±	0.55	1.00		---	1.86	±	0.69	2.14	±	0.69
	Gritty	1.00		---	1.00		---	1.00			1.00		---

† Informal sensory appraisal was accomplished by seven in-house scientists, some formally trained and coaching the session, using a 0 - 3 scale where 0 was no response, 1 = low, 2 = medium and 3 = high. Saltines were used between randomized 3-digit coded samples, as well as fresh water rinsing and expectoration. Averages (± standard deviations) include only positive responses from the 1-3 scale, with 0's (no response) removed.

‡ Acronyms for commercial rice beverages treatments were: CRB#1, not enriched (un-fortified) brown rice (BR) beverage; CRB#2, a "sprouted", unsweetened, fortified brown rice beverage; CRB#3 and CRB#4 were unsweetened, fortified brown rice beverages. Ingredient labels indicated no exogenously added sugar (unless labeling caveats allow inclusion of "rice syrup" or "rice syrup solids" as part of the "brown rice"). The nutrition labels indicated from <1 to 10g sugars. However, soluble solids analyses indicated from 6.0 - 10.8 °Brix (Beaulieu et al., 2020). According to the ingredient labels, no gums like gellan or gum Arabic were added in these commercial samples, and all had added plant oils.

Table 3. Individual γ-aminobutyric acid (mg/g) and phenolics means (GAE, mg/g) in repeated independent experiments assessing the effects of germinating and processing brown Rondo rice into beverages.

	BRR †		GBR		PWM	
**Trial**	**GABA**	**Phenolics**	**GABA**	**Phenolics**	**GABA**	**Phenolics**
1	0.41 ± 0.07	6.55 ± 1.07	0.52 ± 0.10	8.05 ± 0.44	0.39 ± 0.03	9.17 ± 1.17
2	0.36 ± 0.06	7.21 ± 0.48	0.42 ± 0.17	7.52 ± 0.45	0.40 ± 0.01	8.30 ± 0.38
3	0.47 ± 0.12	9.05 ± 2.41	0.53 ± 0.07	*8.29 ± 2.39* ‡	0.39 ± 0.02	7.15 ± 0.40
4	0.41 ± 0.07	7.87 ± 0.90	0.49 ± 0.16	9.38 ± 1.37	0.39 ± 0.06	8.64 ± 2.31

† Acronyms for rice type and treatments are: BRR, brown Rondo rice; GBR, germinated brown rice; PWM, post wet milling; GABA, γ-aminobutyric acid, and phenolics or "total phenolics" by Folin-Ciocalteu method.

‡ Italicized and underlined mean for phenolics indicate an independent trial that did not follow the normal trend whereby GBR > BRR. All averages were from n=3 replicates from independent experimental units, ± standard deviation.

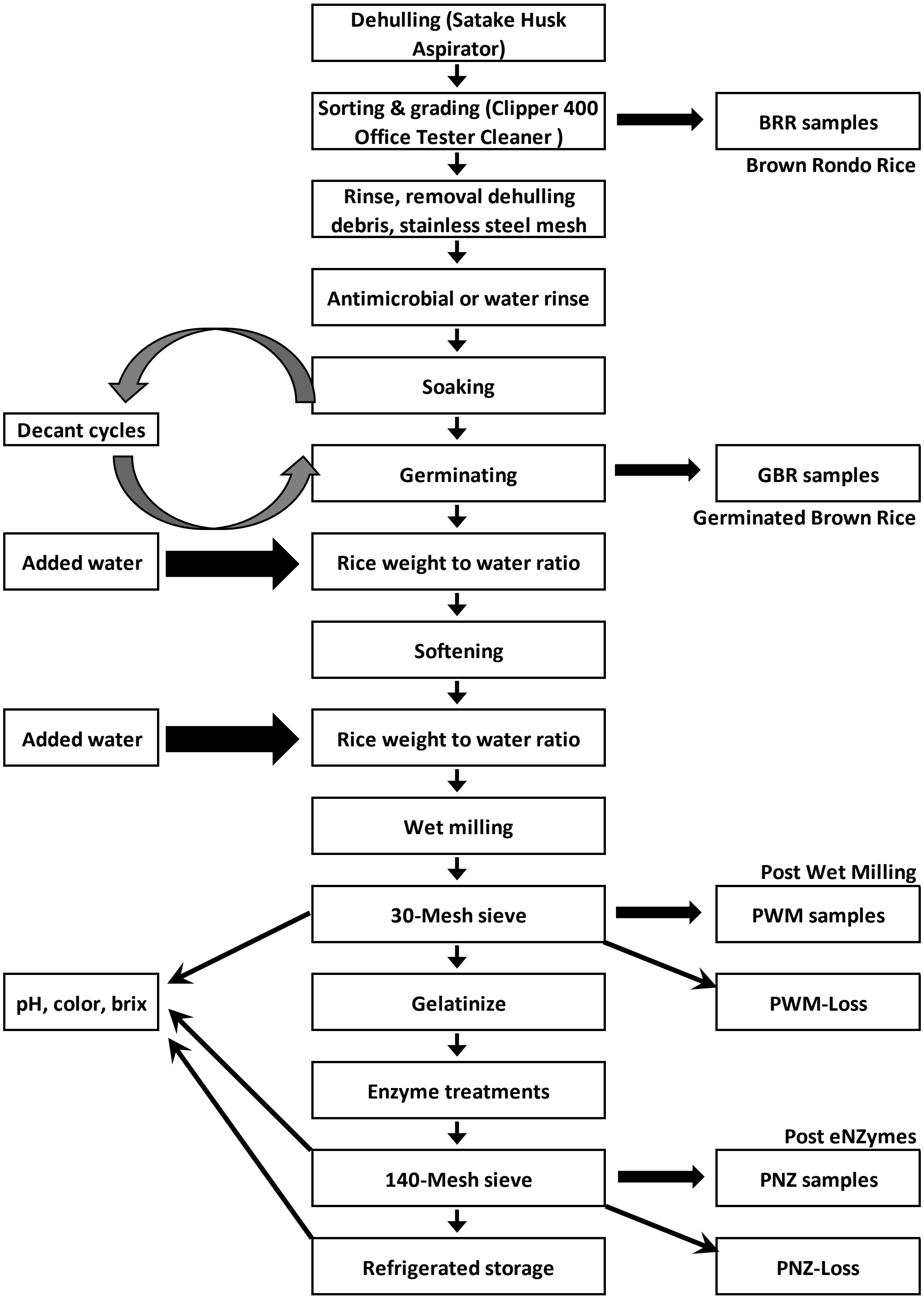

Figure 1. Empirically deduced optimized germinated brown rice "free-flowing" processing protocol and sampling regime. [Previously published, (Beaulieu et al., 2020); https://onlinelibrary.wiley.com/doi/10.1002/fsn3.1534].

Reference

1. Beaulieu J.C., S Reed, J.M. Obando-Ulloa and A.M. McClung. 2020. Green processing protocol for germinating and wet milling brown rice beverage formulations: Sprouting, milling and gelatinization effects. Food Sci & Nutr. 2020(8):2445-2457.
